# On-farm welfare assessment of semi-extensively managed sheep using Animal Welfare Indicators (AWIN) protocol

**DOI:** 10.5194/aab-68-459-2025

**Published:** 2025-07-10

**Authors:** Serdar Koçak, Zehra Bozkurt, Özlem Gücüyener Hacan, Koray Çelikeloğlu, Mustafa Tekerli, Mustafa Demirtaş, Samet Çinkaya, Metin Erdoğan

**Affiliations:** 1 Department of Animal Science, Faculty of Veterinary Medicine, Afyon Kocatepe University, 03200 Afyonkarahisar, Türkiye; 2 Department of Veterinary Biology and Genetics, Faculty of Veterinary Medicine, Afyon Kocatepe University, 03200 Afyonkarahisar, Türkiye

## Abstract

As in the European Union, new sets of animal welfare standards have been implemented in Türkiye, a candidate country for EU membership. However, there is still a limited amount of data available to form the basis of policy measures aimed at improving the welfare of animals in the extensive and semi-extensive sheep production systems. Türkiye's geographical and climatic characteristics at the crossroads of continents influence semi-extensive sheep production. This study was conducted in 56 commercial sheep farms in the Emirdağ district of Afyonkarahisar Province, Türkiye, to assess sheep welfare and to identify key welfare risks using the animal-based assessment protocol of the Animal Welfare Indicators (AWIN) project. Each farm was visited once, and data on the physical conditions of housing and equipment were obtained by measuring or observing, while data on animal management were obtained by interviewing farmers. A total of 702 sheep were individually assessed on the farms as part of the animal-based sheep assessment using the AWIN protocol.

Animal management practices, including providing adequate space per animal (1.7 m^2^), hand milking, veterinary consultation, avoiding painful mutilations in lambs, and bottle-feeding weak newborn lambs, supported sheep welfare. No cases of excessive scratching, myiasis, leg problems, or clinical mastitis were observed. Major body lesions were rare (major lesions were mostly found on the udder at 0.65 % and least on the legs at 0.00 %), while lameness (1.3 %) and hoof overgrowth (6.27 %) were at relatively low levels. No animals exhibited social withdrawal or stereotypical behavior. Poor body condition (30.04 %), severe faecal soiling (36.85 %), wet and soiled fleece (48.65 %), poor wool quality (22.94 %), and respiratory problems (9.30 %) may result from poor air quality, inadequate comfort, and insufficient floor hygiene in barns. Potential welfare risks were identified as follows: milking sheep immediately after birth; negative human–animal interactions; the lack of loading ramps, water, and shade in village pastures, as well as of regular foot care, mastitis and lameness control programs, and farm records. As a result, this study provides insight into the animal welfare of animals on family sheep farms in Inner West Anatolia, Türkiye, and highlights the need for high-quality feed, better housing conditions, and good animal handling. The findings could be used to support policies to improve animal welfare standards, including barn design, animal welfare management, and awareness of animal needs.

## 1 Introduction

Sheep are predominantly reared in extensive and semi-extensive production systems with the primary aim of reducing production costs (Avilez et al., 2021). These systems are particularly widespread in marginal areas, where low-input and extensive systems based on natural vegetation are used (Sevi et al., 2009). Sheep are considered to be highly resilient animals, and this unfounded belief has led to a long period of neglect of the welfare issues related to sheep (Sevi et al., 2009; Molnár, 2022). Previous research in this area has focused on the effects of intensive sheep farming on sheep welfare, similarly to other livestock species (Napolitano et al., 2008; Liu et al., 2012; Moschovas et al., 2021). However, studies investigating welfare issues in extensive and semi-extensive systems have identified some problems, but their extent remains unknown and requires further investigation (Munoz et al., 2019a; Mondragón-Ancelmo et al., 2020).

Recent reforms in the Common Agricultural Policy (CAP) of the European Union are designed to elevate welfare standards in livestock production systems, addressing the growing demands of consumers (Ryland, 2015). In a parallel context, as a candidate country for EU membership, Türkiye's adaptation of relevant policies could lead to input adjustments and improved management practices in extensive and semi-extensive sheep farming systems. The welfare implications of these adjustments and the results should be assessed and incorporated into decision-making (Stott et al., 2005). However, it is necessary to identify, quantify, and conduct risk analyses for different aspects of animal welfare in sustainable sheep farming systems (Stott et al., 2005). The same requirements are necessary for the payment of subsidies (cross-compliance). These are linked to compliance with minimum animal welfare standards as part of EU policy (Stott et al., 2005). Türkiye, which is negotiating to become a full member of the EU, is located where the Middle East, Europe, and Asia meet. Semi-extensive sheep production is well developed in the country due to the suitability of its climate and geography, as well as the socio-economic structure of its society. However, there is almost no research on the welfare of the animals in these sheep farms. There is a need to increase the compliance of sheep farms in Türkiye with CAP policies as this will enhance the competitiveness of semi-extensive sheep farms in the market while simultaneously elevating the welfare levels of the animals. A previous study carried out in Afyonkarahisar, located in the Inner West Anatolia region (where the Aegean and Inner Anatolian regions meet), one of the regions with the highest sheep population density in the country, provided some preliminary results on the conditions on these farms (Kılıç et al., 2013).

The aim of this study was to assess the welfare of sheep and to identify key welfare risks in semi-extensively commercial sheep farms in the Afyonkarahisar Province of Türkiye using the AWIN animal-based assessment protocol.

## 2 Material and methods

### 2.1 Animals, sampling, and farm characteristics

This study was conducted in the province of Afyonkarahisar, located in the Inner Western Anatolia region of Türkiye, where semi-intensive sheep farming is common, with the dominant breed being the indigenous Pırlak sheep. Due to time and cost constraints, a multi-stage cluster sampling technique was used, focusing on the district of Emirdağ, which is one of the districts with a high concentration of Pırlak sheep farming in the province. The study was carried out in autumn season (October and November) and on 56 farms located in the village of Yüreğil (latitude: 39°1^′^31.82^′′^ N, longitude: 31°1^′^29.99^′′^ E), where only Pırlak sheep are reared. A formula ( n= N.P.Q.Z α ${}^{2}/((N-1)\cdot d^{2}+$ P.Q.Z $\alpha^{2}$ )) was used to determine the sample size in the district of Emirdağ, with the following parameters: population size ( N ) = 594; probability values as percentages ( P and Q ), with P= 50 % and Q= 50 %; theoretical value Zα= 1.96 at the significance level; and sampling error or effect size d= 6 %. The farms visited represent about 10 % of the Pırlak farms in the district of Emirdağ, with no other animal species being reared (Bozkurt et al., 2023). A preliminary interview was conducted with all sheep farmers in the village of Yüreğil whose farms were active at the time of the visit with regard to the research objectives, animal welfare protocol, and the activities of the assessors. The research was carried out on sheep farms where the owners had agreed to participate in the research (AWIN, 2015). To collect data on farm characteristics and management practices, farmers were interviewed using a questionnaire containing 36 questions, which were classified into four sections: livestock; housing and feeding; health and care; and husbandry practices that encompassed breeding, milking, shearing, marketing, lamb care, transport, and farm records. The following parameters were carefully evaluated in order to analyze the specific characteristics of the Pırlak sheep farms: flock size (number of ewes, rams, and lambs under 6 months of age per farm), main farming purposes, and the type of farming system used. The current status of farm management practices that could potentially affect the welfare of sheep on farms (such as the presence of written health and welfare plans and regular controls of animal health and hoof care) was recorded. We also collected data on mutilation procedures in lambs (castration, tail docking, dehorning), milking practices (timing of first milking after lambing, methods for maintaining udder hygiene and health, milking type), breeding practices (breeding program and sire selection), care of the lambs (supplementary feeding of the lambs and cross-fostering of orphaned lambs), keeping farm records (births, mortalities, production), and the presence of loading and unloading ramps on the farm.

### 2.2 Animal-based indicators for the assessment of sheep welfare

The animal-based welfare assessment was carried out using the second-level indicators of the AWIN welfare assessment protocol for sheep (AWIN, 2015; Caroprese et al., 2016). On each farm, sheep were randomly selected by assessors and placed in separate pens. The number of animals sampled on each farm was determined by the number of adult ewes (AWIN, 2015). On average, 24.86 % of the flock was sampled (ranging from 6.52 % to 50.00 %) based on criteria such as farm conditions (whether all sheep are kept together), the farmer's permission (whether assessor is allowed to handle the number of sheep requested), and the suitability of the animals for handling (whether animals have a fragile structure or a fearful temperament, which may cause them to react with excessive fear or overreaction during the assessment). The second-level welfare assessment consisted of scoring body condition score (BCS), fleece cleanliness, fleece quality, excessive itching, faecal soiling, respiratory quality, ear tag availability, body lesions (head and/or neck, ear, eyes, body, udder, tail), clinical mastitis, hoof overgrowth, tail length, leg injuries, and lameness. The welfare assessment protocol on each farm was applied by two assessors: one trained and experienced in farm animal welfare assessment and the other trained by this assessor prior to the study with theoretical and practical sessions on the AWIN welfare assessment protocol for sheep in semi-intensive production systems. After the sheep were placed in a pen suitable for handling and inspection, the same two assessors carried out individual assessments by observing the animals from both the front and back (AWIN, 2015). The criteria that were used for the scoring of these indicators are described in Table 1. A total of 702 animals were assessed on the farms within the framework of the second-level welfare assessment of sheep. The sheep (%) distribution in the scores for each welfare indicator was determined on each farm. For the Familiar Human Approach Tests (FHATs), farmers were asked to enter the barn and approach the animals calmly and randomly, as they would during their daily care routine. An assessor, standing quietly away from the sheep, observed the sheep's reactions to the farmer and scored them based on whether they allowed the farmer to make contact. The number and percentage (%) of animals assigned to each scoring group were determined (AWIN, 2015).

**Table 1:** AWIN recommendations for animal-based welfare assessment.

Parameters	Indicator criteria	Scores
Body condition score (BCS)	0 %–100 % of scores	Score 0: thin ( < 2.0 BCS); score 1: good ( > 2.0, < 4.0 BCS); score 2: fat ( > 4.0 BCS)
Fleece cleanliness	0 %–100 % of scores	Score 0: clean and dry, fleece shows no sign of dirt or contamination Score 1: dry or slightly damp due to current weather conditions, slight mud or dirt on body attributed to handling or pen from that day (handled animals) Score 2: very damp or wet, coat contaminated by mud or dung from fields or hill Score 3: very wet, very heavily soiled with mud or dung
Fleece quality	0 %–100 % of scores	Score 0: good fleece quality; score 1: slight fleece loss (less than 10 cm^2^ area); score 2: extensive fleece loss (more than 10 cm^2^ area)
Excessive itching	(% itching or scratching)	Proportion of the animals showing signs of excessive itching
Faecal soiling	0 %–100 % of scores	Score 0: no present faecal soiling; score 1: very light soiling (a small quantity of faecal matter in the wool around the anus); score 2: light soiling and dags around the anus and dags (matted areas of faecal matter adhering to the wool) in this area only; score 3: soiling and dags extending beyond the anus to the tail and onto the upper part of the legs; score 4: extensive soiling with dags extending down the legs as far as the hocks
Respiration quality	0 %–100 % of scores	Score 0: normal respiration; score 1: presence of a respiratory problem such as panting
Ear lesions	0 %–100 % of scores	Score 0: no lesion; score 1: minor lesions (rips or tears associated with loss of tags); score 2: major lesions (scars, marks, or incisions, healed or unhealed)
Ear tag	0 %–100 % of scores	Score 0: ear tag was available; score 1: ear tag was not available
Eye lesions	0 %–100 % of scores	Score 0: no lesions; score 1: minor lesions (eye discharge, mucosal hyperemia, etc.); score 2: major lesions (eye injuries, exophthalmos, etc.).
Udder lesions	0 %–100 % of scores	Score 0: no; score 1: yes (small or major skin lesions, etc.)
Clinical mastitis	0 %–100 % of scores	Score 0: no; score 1: yes
Stocking density	m^2^ per head	m^2^ space allowance per head on paddock area
Head and/or neck lesions	0 %–100 % of scores	Score 0: no lesions; score 1: minor lesions (hairless patches or scratches, healed lesions, open wounds not penetrating the muscle layer, 2–10 cm in size); score 2: major lesions (open wounds more than 10 cm in length and penetrating the muscle layer in terms of depth)
Body lesions	0 %–100 % of scores	Score 0: no lesions; score 1: minor lesions (hairless patches or scratches, healed lesions, open wounds not penetrating the muscle layer, 2–10 cm in size); score 2: major lesions (open wounds more than 10 cm in length and penetrating the muscle layer in terms of depth)
Tail length	0 %–100 % of scores	Score 0: % full tails; score 1: tail docked

**Table 1:** Continued.

Parameters	Indicator criteria	Scores
Tail lesions	0 %–100 % of scores	Score 0: no lesions; score 1: minor lesions (hairless patches or scratches, healed lesions, open wounds not penetrating the muscle layer, 2–10 cm in size); score 2: major lesions (open wounds more than 10 cm in length and penetrating the muscle layer in terms of depth)
Leg injuries and lesions	0 %–100 % of scores	Score 1: no lesions; score 2: minor lesions (there were calluses, hairless patches, or shallow injuries on the legs); score 3: major lesions (there were open wounds on the legs and swelling on the joints of the legs)
Lameness	0 %–100 % of scores	0: not lame; 1: minor lameness; 2: lame; 3: severe lameness
Hoof overgrowth		Score 0: hooves of appropriate length and shape; score 1: hooves are overgrown
Social withdrawal	(% showing social withdrawal)	Proportion of animals showing signs of social withdrawal
Stereotypy	(% showing stereotypical behavior)	Proportion of animals showing signs of stereotypical behavior
Familiar human approach test (FHAT)	0 %–100 % of scores	Score 0: sheep voluntarily interacted with farmer; score 1: sheep did not avoid the familiar human's approach but did not interact voluntarily with the farmer; score 2: sheep did avoid the familiar human's approach

### 2.3 Resource-based indicators and behavioral observations for the assessment of sheep welfare

The observation, measurement, and assessment form included 16 parameters designed to determine farm characteristics and to measure the resource-based welfare of the animals. This form incorporated resource-based welfare indicators and simple, rapid observations of animal behavior. The welfare parameters assessed by this form and the farmer questionnaire have been developed using an approach consistent with the WQ^®^ Animal Welfare Principles, drawing on the resource-based welfare indicators for the AWIN first-level welfare assessment and knowledge gained from previous studies (Goddard, 2011; van Eerdenburg et al., 2018; Mondragón-Ancelmo et al., 2020; Nenadović et al., 2020; Bodas et al., 2021; Toro-Mujica and Riveros, 2021; Richmond et al. 2017). Farms were assessed according to four welfare principles: good housing (including type of housing, penning, shade, lighting, bedding, cleaning and hygiene, quality of ventilation and flooring in barns, and protection from predators in pastures), good feeding (traits of feeders and drinkers and provision of feed and water), good health (responsibility for treating sick animals and use of medication, parasite infestations in the previous year, and scanning pregnant ewes for determination of litter size), and appropriate behavior (presence of tethered or isolated animals on farms, grazing, and distance between barn and pasture). Farms were given a score of 0 (no or absent) or 1 (yes or present) for each parameter examined (two-point-scale scoring) (AWIN, 2015; Marcone et al. 2022). In addition, the parameters of social withdrawal and stereotypy behavior were administered as simple and rapid behavioral assessments as part of the first-level welfare assessment of the AWIN protocol for sheep. An assessor stood quietly away from the sheep in the barn for at least 5 min. The assessor was careful not to attract the attention of the sheep until they had lost the interest of the sheep. The sheep were observed for 20 min for signs of social withdrawal (standing apart from the social group and being unresponsive to activity around them) and stereotypical behavior (repeated aimless movements such as turning the head back over the shoulders, looking up, or pulling the wool from another sheep's back). The total time taken to complete the farmer questionnaire and to take measurements for the resource-based welfare indicators was between 45 and 60 min for each farm. The other findings of this research regarding the management of housing, feeding, reproduction, animal health, milking, shearing, and marketing, together with farm assets, inputs, and storage, have been presented in a separate paper (Bozkurt et al., 2023).

### 2.4 Statistical analyses

Descriptive statistics were calculated using data on farm characteristics and resource-based and animal-based welfare parameters (mean, standard error (SEM), median, minimum, maximum, 25 % and 75 % quartile values). SPSS 21 software for Windows and Microsoft Excel packages were used for the statistical analyses of the parameters studied.

## 3 Results

The characteristics of the visited sheep farms are given in Table 2. The average numbers of ewes, rams, and lambs in farms were determined to be 79.78, 2.05, and 96.33, respectively. All the farms adopted dual-purpose production, mainly focusing on milk and benefiting from the common village pasture. In semi-extensive farms, sheep were housed in enclosed barns throughout the year and during the night. All farms had traditional barnyards enclosed by stone walls or a wooden fence, while some farms added shading to these barnyards, providing notable improvements in functionality and comfort. Among the farm management parameters assessed, it was found that there were no written herd health and welfare plans, no regular veterinary checks, no foot care plans, and no farm records. On these farms, there were no ramps for the loading and unloading of the animals. The farmers have chosen not to engage in certain practices, including the use of breeding programs and artificial insemination; the use of machine milking; and the performance of mutilations in lambs such as castration, tail docking, and dehorning. They also start milking immediately after lambing ( 0.82±0.05 ). While Pırlak farmers are less concerned about udder cleaning before and after milking, it was noteworthy that some farmers apply antibiotics to the udder during the dry period ( 0.07±0⋅04 ). On the majority of the farms, lambs that were born weak or that were born from multiple lambings were fed with a supplement of natural milk from a bottle in the first few days after birth ( 0.75±0.06 ). A few farms used cross-fostering for orphaned lambs ( 0.09±0.04 ).

**Table 2:** Characteristics of the sheep farms.

Parameters		Mean	SEM	Median	Min	Q25	Q75	Max
Animals (heads per farm)	Ewe	79.78	6.74	63.00	20	40.00	120.00	228.00
	Ram	2.05	0.36	1.00	1.00	1.00	2.00	20.00
	Lamb	96.33	8.18	80.00	27	48.00	135.00	275.00
Main purpose	Dairy	0.82	0.05	1.00	0.00	1.00	1.00	1.00
	Meat	0.13	0.04	0.00	0.00	0.00	0.00	1.00
	Meat and dairy	0.05	0.03	0.00	0.00	0.00	0.00	1.00
Farming type	Farming system type (semi-extensive)	1.00	0.00	1.00	1.00	1.00	1.00	1.00
Farm management								
	Written flock health and/or welfare Plans (0 = no; 1 = yes)	0.00	0.00	0.00	0.00	0.00	0.00	0.00
	Regular veterinary controls (0 = no; 1 = yes)	0.00	0.00	0.00	0.00	0.00	0.00	0.00
	Regular foot care (0 = no; 1 = yes)	0.00	0.00	0.00	0.00	0.00	0.00	0.00
	Castration (0 = no; 1 = yes)	0.00	0.00	0.00	0.00	0.00	0.00	0.00
	Tail docking (0 = no; 1 = yes)	0.02	0.02	0.00	0.00	0.00	0.00	1.00
	Dehorning (0 = no; 1 = yes)	0.00	0.00	0.00	0.00	0.00	0.00	0.00
Milking	Does milking start right after lambing? (0 = no; 1 = yes)	0.82	0.05	1.00	0.00	1.00	1.00	1.00
	Machine milking (0 = no; 1 = yes)	0.00	0.00	0.00	0.00	0.00	0.00	0.00
	Udder cleaning before and/or after milking (0 = no; 1 = yes)	0.00	0.00	0.00	0.00	0.00	0.00	0.00
	Antibiotic application into the breast in the dry period (0 = no; 1 = yes)	0.07	0.04	0.00	0.00	0.00	0.00	1.00
Breeding	Sheep breeding scheme employed (0 = no; 1 = yes)	0.00	0.00	0.00	0.00	0.00	0.00	0.00
	Artificial insemination (0 = no; 1 = yes)	0.00	0.00	0.00	0.00	0.00	0.00	0.00
Lamb care	Supplementary lamb feeding with feeding bottles (0 = no; 1 = yes)	0.75	0.06	1.00	0.00	0.25	1.00	1.00
	Cross-fostering for orphaned lambs (0 = no; 1 = yes)	0.09	0.04	0.00	0.00	0.00	0.00	1.00
Farm records	Sheep mortality records (0 = no; 1 = yes)	0.00	0.00	0.00	0.00	0.00	0.00	0.00
	Lamb birth and death records (0 = no; 1 = yes)	0.04	0.03	0.00	0.00	0.00	0.00	1.00
	Production records (0 = no; 1 = yes)	0.00	0.00	0.00	0.00	0.00	0.00	0.00
Transport	Animal loading and unloading ramps (0 = no; 1 = yes)	0.00	0.00	0.00	0.00	0.00	0.00	0.00

**Table 3:** Results of animal-based indicators in the second-level AWIN welfare assessment for sheep.

Welfare indicators		Mean	SEM	Median	Min	Q25	Q75	Max.
Animal sampling	Animal sampling ratio (%)	24.86	1.88	24.04	6.52	12.88	33.93	50.00
	Animal sampling number (head)	14.44	0.32	15.00	10.00	15.00	15.00	25.00
BCS (%)	Thin	30.04	2.70	23.33	6.25	18.33	37.27	86.67
	Good	68.26	2.74	73.33	13.33	60.00	80.00	93.70
	Fat	1.70	0.55	0.00	0.00	0.00	0.00	20.00
Fleece cleanliness (%)	Clean and dry	10.45	2.80	0.00	0.00	0.00	13.33	90.00
	Slightly damp, mud or dirt	40.90	4.00	33.33	0.00	13.33	67.19	90.00
	Very wet, contaminated by mud and dung	38.32	4.02	29.00	0.00	13.33	60.00	93.33
	Very wet and heavily soiled with mud or dung	10.33	2.80	0.00	0.00	0.00	10.83	80.00
Fleece quality (%)	Good	77.06	2.65	80.00	13.33	66.67	88.13	100.00
	Slight fleece loss	19.36	2.05	20.00	0.00	9.58	27.50	66.67
	Extensive fleece loss	3.58	1.33	0.00	0.00	0.00	6.67	60.00
Excessive itching (%)	Yes	0.00	0.00	0.00	0.00	0.00	0.00	0.00
Faecal soiling (%)	Not present	9.93	2.67	0.00	0.00	0.00	10.83	86.67
	Very light soiling around the anus	22.51	3.75	13.33	0.00	6.67	29.33	93.75
	Light soiling and dags around the anus	30.71	3.36	26.67	0.00	13.33	50.00	80.00
	Soiling and dags extending beyond anus to tail and thigh area	26.37	3.73	20.00	0.00	4.99	33.33	86.67
	Extensive soiling and faecal dags down to the tail, thighs, and shins	10.48	2.12	5.33	0.00	0.00	13.33	58.33
Respiration quality (%)	No respiratory issues	90.70	2.70	100.00	30.00	94.50	100.00	100.00
	Respiratory problems	9.30	2.70	0.00	0.00	0.00	5.50	70.00
Ear lesions (%)	No lesions	90.93	2.05	100.00	40.00	85.83	100.00	100.00
	Minor lesions	8.52	1.95	0.00	0.00	0.00	13.31	50.00
	Major lesions	0.55	0.32	0.00	0.00	0.00	0.00	10.00
Ear tag (%)	Available	94.65	1.71	100.00	40.00	93.33	100.00	100.00
	Not available	5,35	1.72	0.00	0.00	0.00	6.67	60.00
Eyes lesion (%)	No	98.20	0.53	100.00	83.33	100.00	100.00	100.00
	Minor	1.50	0.45	0.00	0.00	0.00	0.00	10.00
	Major	0.30	0.21	0.00	0.00	0.00	0.00	8.34
Udder lesions (%)	No	99.35	0.33	100.00	87.50	100.00	100.00	100.00
	Yes	0.65	0.33	0.00	0.00	0.00	0.00	12.50
Evidence of clinical mastitis	Yes	0.00	0.00	0.00	0.00	0.00	0.00	0.00
Head and/or neck lesions (%)	No lesions	98.54	0.65	100.00	75.00	100.00	100.00	100.00
	Minor lesions	1.29	0.54	0.00	0.00	0.00	0.00	16.70
	Major lesions	0.17	0.17	0.00	0.00	0.00	0.00	8.30
Body lesions (%)	No lesions	98.95	0.45	100.00	8.33	100.00	100.00	100.00
	Minor lesions	0.72	0.39	0.00	0.00	0.00	0.00	16.67
	Major lesions	0.33	0.24	0.00	0.00	0.00	0.00	10.00
Tail length (%)	Tail docked	0.13	0.13	0.00	0.00	0.00	0.00	6.70
Tail lesion (%)	No	99.46	0.26	100.00	93.30	100.00	100.00	100.00
	Yes	0.54	0.26	0.00	0.00	0.00	0.00	6.70
	Myiasis (flystrike)	0.00	0.00	0.00	0.00	0.00	0.00	0.00

**Table 3:** Continued.

Welfare indicators		Mean	SEM	Median	Min	Q25	Q75	Max.
Leg injuries and lesions (%)	No lesions	98.74	0.46	100.00	86.67	100.00	100.00	100.00
	Minor lesions	1.26	0.46	0.00	0.00	0.00	0.00	13.33
	Major lesions	0.00	0.00	0.00	0.00	0.00	0.00	0.00
Lameness (%)	Not lame	93.51	1.21	93.54	66.67	91.50	100.00	100.00
	Minor lameness	5.19	1.15	0.00	0.00	0.00	6.70	30.00
	Lame	0.90	0.35	0.00	0.00	0.00	0.00	10.00
	Severe lameness	0.40	0.23	0.00	0.00	0.00	0.00	6.70
Hoof overgrowth (%)	Appropriate or groomed	93.73	1.72	100.00	53.30	93.32	100.00	100.00
	Hooves were overgrown	6.27	1.72	0.00	0.00	0.00	6.68	46.70
FHAT (%)	Sheep voluntarily interacting with farmer	11.78	2.02	5.00	0.00	0.00	20.00	60.00
	Sheep did not avoid human approach but did not interact voluntarily with the farmer	31.11	3.72	25.00	5.00	10.00	40.00	90.00
	Sheep did avoid the familiar human's approach	57.11	4.74	70.00	0.00	30.00	87.50	95.00

The animal-based welfare indicators for the second level of the AWIN welfare assessment are presented in Table 3, together with the results (%) of the individual sheep scored. The mean proportions of sheep scored as fat and thin in terms of body condition were 1.70±0.55 % and 30.04±2.70 %, respectively. The proportions of sheep scored as slightly dirty; wet and dirty; and wet, dirty, and heavily soiled in terms of fleece cleanliness were 40.90±4.00 %, 38.32±4.02 %, and 10.33±2.80 %, respectively. The mean ratios of sheep with slight or extensive fleece loss detected were 19.36±2.05 % and 3.58±1.33 %. On the farms visited, no animals were observed showing signs of excessive itching. The mean rates of sheep with regard to the levels of soiling around the anus (very light soiling, light soiling with dags, soiling with dags, and extensive soiling with faecal dags down to the tail) were 22.51±3.75 %, 30.71±3.36 %, 26.37±3.73 %, and 10.48±2.12 %, respectively. The mean proportion of sheep with respiratory problems was 9.30±2.70 %. The average proportions of minor and major lesions on the ears, eyes, head and/or neck, body, and legs of the animals were 8.52±1.95 and 0.55±0.32 , 1.50±0.45 and 0.30±0.21 , 1.29±0.54 and 0.17±0.17 , 0.72±0.39 and 0.33±0.24 , and 1.26±0.46 and 0.00±00.0 . The mean percentages of sheep classified as minorly lame, lame, and severely lame were 5.19±1.15 , 0.90±0.35 , and 0.40±0.23 %, respectively. The mean proportion of sheep scored as having overgrown hooves was 6.27±1.72 %. According to the results of the familiar human approach test, the ratios of sheep interacting voluntarily with the farmer, not avoiding the familiar human's approach but not interacting voluntarily with the farmer, and avoiding the familiar human's approach were 11.78±2.02 , 31.11±3.72 , and 57.11±4.74 %, respectively.

The results of the resource-based welfare assessment on the Pırlak sheep farms are presented in Table 4. On the farms, which provided overnight housing throughout the year, adequate lighting was provided in all of the barns. However, ventilation, supply of dry bedding or a dry lying area, and cleaning and hygiene of the barns were inadequate generally, and the barn floors were wet and slippery. All farms provided animals with protection from predators (through shepherd and guard dog supervision) when animals were in the pasture, but very few farms provided shade. The heights of the feeders and drinkers were 43.45±0.54 and 39.87±1.15 cm, respectively. These feeders were functional and suitable for feed intake, and, on many farms, feed and water were not available during the welfare assessment. In most farms, sick animals were treated by the farmer or owner after consultation with a veterinarian ( 0.88±0.05 ). However, farms did not scan the pregnant ewes to determine litter size. The numbers of ewes reformed and experiencing dystocia were 5.45±1.09 and 2.29±0.44 , respectively. The average farm score was 0.04±0.03 for parasite infestation detected in the previous year. To determine whether the behavioral needs of the animals were being met, resource-based animal welfare parameters were examined. No tethered, isolated, socially withdrawn, or stereotyped behavior by animals was observed on the farms. There were no artificial or fenced pastures on any of the farms, but sheep were grazed on common village pastures at an average distance of 5.63±0.27 km from the farm.

**Table 4:** The results of resource-based animal welfare indicators and animal behavior measurements carried out within the first level of animal welfare assessment.

Parameters		Mean	SEM	Median	Min	Q25	Q75	Max
Good housing	Overnight housing year-round (0 = no; 1 = yes)	1.00	0.00	1.00	1.00	1.00	1.00	1.00
	Ventilation (3: good; 2: partly; 1: insufficient)	1.82	0.07	2.00	1.00	2.00	2.00	3.00
	Access to shade in farmyard (0 = not present; 1 = present)	0.16	0.05	0.00	0.00	0.00	0.00	1.00
	Access to water and artificial shade on the pasture (0 = not present; 1 = present)	0.07	0.04	0.00	00.0	0.00	0.00	1.00
	Protected from predators in the pasture (0 = no; 1 = yes)	1.00	0.00	1.00	1.00	1.00	1.00	1.00
	Adequate lighting (minimum of 8 h d^−1^) (0 = no; 1 = yes)	1.00	0.00	1.00	1.00	1.00	1.00	1.00
	The floor is wet and slippery (0 = no; 1 = yes)	0.88	0.05	1.00	0.00	1.00	1.00	1.00
	Dry bedding or dry lying area for all animals (0 = no; 1 = yes)	0.00	0.00	0.00	0.00	0.00	0.00	0.00
	Barn cleaning and hygiene (3: good; 2: moderate; 1: poor)	1.21	0.07	1.00	1.00	1.00	1.00	3.00
Good feeding	Feeder height (cm)	43.45	0.54	45.00	34.00	42.00	45.00	59.0
	The presence of feed in the feeders during the evaluation (0 = no; 1 = yes)	0.13	0.05	0.00	0.00	0.00	0.00	1.00
	Feeders are functional (0 = no; 1 = yes)	1.00	0.00	1.00	1.00	1.00	1.00	1.00
	Feeders are suitable for feed intake (0 = no; 1 = yes)	1.00	0.00	1.00	1.00	1.00	1.00	1.00
	Drinker height (cm)	39.87	1.15	40.00	17.00	35.00	45.00	68.00
	The presence of water in the drinkers during the evaluation (0 = no; 1 = yes)	0.55	0.07	1.00	0.00	0.00	1.00	1.00
	Drinkers are functional (0 = no; 1 = yes)	1.00	0.00	1.00	1.00	1.00	1.00	1.00
	Drinkers are suitable for water intake (0 = no; 1 = yes)	1.00	0.00	1.00	1.00	1.00	1.00	1.00
Good health	Who is doing the treatments?							
	Veterinarian (0 = no; 1 = yes)	0.04	0.03	0.00	0.00	0.00	0.00	1.00
	Farmer or owner (0 = no; 1 = yes)	0.96	0.03	1.00	0.00	1.00	1.00	1.00
	Is the veterinarian's advice sought before administering medicines? (0 = no; 1 = yes)	0.88	0.05	1.00	0.00	1.00	1.00	1.00
	Number of reforming ewes (heads)	5.45	1.09	4.00	1.00	3.00	9.00	12.00
	Number of ewes experiencing dystocia (heads)	2.29	0.44	2.00	1.00	1.00	3.25	6.00
	Parasitics diseases in the past 1 year (0 = no; 1 = yes)	0.04	0.03	0.00	0.00	0.00	0.00	1.00
	Are ewes scanned to determine litter size? (0 = no; 1 = yes)	0.00	0.00	0.00	0.00	0.00	0.00	0.00
Appropriate behavior	Presence of tethered or isolated animals (0 = no; 1 = yes)	0.00	0.00	0.00	0.00	0.00	0.00	0.00
	Grazing on village pasture (0 = no; 1 = yes)	1.00	0.00	1.00	1.00	1.00	1.00	100
	Artificial and fenced grassland (0 = not present; 1 = present)	0.00	0.00	0.00	0.00	0.00	0.00	0.00
	Distance between barn and pasture (km)	5.63	0.27	5.00	1.00	5.00	6.00	12.00
Stocking density	Space allowance indoors (m^2^ per head)	1.72	0.16	1.40	0.40	0.93	1.99	6.21
Social withdrawal (%)	(% with social withdrawal)	0.00	0.00	0.00	0.00	0.00	0.00	0.00
Stereotypy (%)	(% with stereotypical behavior)	0.00	0.00	0.00	0.00	0.00	0.00	0.00

## 4 Discussion

Only Pırlak sheep (local sheep breed) were kept on the farms visited during the study. The farms were generally family-run and small to medium sized. The Pırlak sheep is a crossbreed commonly found in Türkiye's western provinces, derived from combining Dağlıç and Kıvırcık breeds (Akçapınar, 1994). The Pırlak sheep is a dual-purpose breed for milk and meat. Most of the farmers indicated that their main purpose was milk production. In fact, the sheep's milk that was left over after consumption by the family was sold as traditional yogurt and cheese and provided an income for the family. Milk production is the basis of the family's livelihood (Bozkurt et al., 2023). These farms milked sheep by hand rather than by using machine milking. There was no specific udder hygiene or protection routine during milking, and there was no mastitis control program. However, clinical mastitis was not detected on these farms by means of the animal-based assessment. Although this is a positive outcome for sheep welfare, many of the farms visited started milking immediately after lambing (82 %). To prevent the risk of lambs not receiving sufficient maternal milk and to avoid potentially poor lamb welfare, it is recommended that flock bio-security measures be strengthened. The artificial feeding of motherless lambs or lambs from multiple births with ewe's milk has been evaluated as a survival aid, especially in the case of weak lambs, particularly given that lambs need their mothers for both social and, more importantly, nutritional support (Orgeur et al., 1998; Napolitano et al., 2008).

It was also positive for lamb welfare that farms do not use practices such as castration, tail docking, and dehorning of lambs. As reported by Windsor and Lomax (2013) and Windsor et al. (2016), not performing surgical modifications such as castration, dehorning, and tail docking on lambs may have a positive effect on lamb welfare as these procedures cause acute pain in lambs. There were no professional animal management plans on the farms (such as sheep breeding plans, artificial insemination, written flock health and welfare plans or recording of lamb births, or mortality or production data). This situation was not surprising as these farms had low investment and economic capacity. On the contrary, Bodas et al. (2021) reported that, although most sheep farms in Europe with different farming systems have written health plans, there are still farms where not all plans are documented. In addition, the farmers surveyed in our study had no training in animal husbandry, health, or welfare and were running sheep farming as a family business. They said their grandparents taught them sheep farming. The same sheep farm managers have some practical knowledge of traditional sheep farming but lack understanding and knowledge of animal welfare management (Bozkurt et al., 2018). Similarly, Bozkurt et al. (2023) argued that the management strategies of Pırlak sheep farms could be supported by increased financial support from the government and by providing farmers with training in sheep breeding, animal health and welfare, and farm management. Parasitic diseases reported by farmers in the last year were low (0.04 %). However, it is noteworthy that these farms had no animal health or welfare programs, and no regular veterinary inspections were carried out. In addition, the lack of regular recording of animal health, productivity, and reproductive processes is a significant gap in ensuring the sustainability of animal welfare. These findings suggest that farmers may lack the necessary knowledge and skills to comply with higher animal welfare standards, which is in line with the findings of Bozkurt et al. (2018). Munoz et al. (2019a) reported that farmers' management practices can influence animal welfare outcomes and that targeted training programs may be an opportunity to change farmers' management attitudes, resulting in improvements in sheep welfare. The lack of a ramp for the loading and unloading of animals on the Pırlak farms can have a negative impact on the welfare of the sheep. This situation can result in the covers of transport vehicles being used as a makeshift ramp. Alternatively, animals can be caught by holding their horns or fleece and being carried onto vehicles. Both of these possibilities can cause varying degrees of distress and fear for the animals (Carnovale et al., 2021). Handling during transport, such as lifting or dragging sheep by their fleece, legs, or horns, can cause pain and discomfort to the animals, and it also increases the risk of horns breaking (Dwyer and Lawrence, 2008).

In this study, the assessment of the principle of appropriate housing in the Pırlak farms was based on the assumption that the enclosed area per animal on the farms would not lead to overcrowding and, thus, to a loss of welfare. This was because Caroprese et al. (2009) reported that housing with a density of 1.5 m^2^ of indoor space per animal did not impact animal welfare. The indoor stocking density was 1.72 m^2^ per head in this study, which is higher than the 1.2 m^2^ per head reported by Stubsjøen et al. (2022) for sheep in Norway. Similar results have been reported for lamb-fattening farms in European countries (Bodas et al., 2021). In addition, this situation may result from farms acquiring fewer animals than their capacity allows due to limited financial resources. Allocated space per animal, both outdoor and indoor, affects the milk yield, behavior, activity, and welfare of sheep (Caroprese et al., 2009). Sheep were provided with at least 8 h of light a day, but the ventilation of the sheep barn was inadequate. It was suggested that these results may be related to the poor ventilation capacity of the traditional barn as the median prevalence of ventilation was recorded as “partly”, indicating the presence of either slight or strong odors in these barns. Resource-based welfare assessments such as ventilation quality were supported by animal-based welfare assessments as respiratory problems were found in 9.30 % of animals. Bozkurt et al. (2023) detected that the barns were built of rubble stone or mixed materials (stone, wood, and adobe) on those farms. In addition, it has been observed that barns' flooring and bedding conditions may harm the sheep's welfare. Wet and dirty floors and inadequate dry bedding were found in the barns. Faerevik et al. (2005) reported that the amount of time sheep spent lying down on hard floors was reduced when straw bedding was used. The proportion of sheep scored as very wet, contaminated, and heavily soiled with mud and dung (48.65 %) for fleece cleanliness in the individual welfare assessments was already high, and these results show that, even if they are standing for a long time, they might be tired and could need to rest on this poor flooring. The degree of fleece dirtiness was reported to be 39.73 % in dairy sheep and 26.53 % in dual-purpose sheep in a study conducted in Italy (Marcone et al., 2022). Fleece dirtiness can be influenced by various factors, such as whether sheep have been lying in wet or muddy areas (Richmond et al., 2017), the prevalence of diarrhoea, and climatic conditions (Hadley et al., 1997). Fleece dirtiness points to poor welfare standards in terms of both these enterprises and good housing principles as it indicates the extent to which sheep are contaminated by external sources such as rain, mud, and dirty pens (Richmond et al., 2017). All farms had open yards where the sheep could move freely, but only 16 % of farms had artificial shelters to provide shade in these yards. Similarly, there were no artificial shelters in the village pasture where the animals grazed. The village pasture with small tree communities (shrubs, oaks, junipers, etc.) provided limited natural shade for the sheep. However, there was no access to water in the village pasture, especially during the long dry months. Under these grazing conditions, it is thought that the sheep grazing primarily on the pasture were exposed to heat stress and dehydration during all-day grazing. Silanikove (2000) reported that high ambient temperatures and indirect solar radiation are environmental stressors putting pressure on animals. Liu et al. (2012) reported that providing shade during the grazing period reduced stress in sheep reared under high temperatures. It has therefore been argued that night grazing for sheep, particularly during hot periods, would be beneficial both in terms of protection from thermal stress and for nutrition. However, the lack of water resources within the pasture indicated that sheep could be without water for hours between departure from and return to the barn. Silanikove (2000) also emphasized the importance of ensuring that sheep had access to water at least once during the grazing period. Source-based assessments of the principle of good feeding have shown that the standards for feeders and drinkers (height and functionality of feeders and drinkers and suitability for feed and water intake) on farms do not have a negative impact on animal welfare. Almost one-third of the ewe flocks were found to be thin in terms of body condition score, which is an indication that the sheep feeding standards on the Pırlak farms were low. The percentage of good body condition scores in sheep was 68.26 %. This percentage is very close to that reported by Battini et al. (2021) for semi-extensive goats ( 67.9±5.7 %). This suggests that pasture capacity may be insufficient as the farms were mainly pasture-based. In addition, the use of concentrate feed was found to be insufficient. Bozkurt et al. (2023) reported that 76.79 % of these farms purchased feed. It is assumed that these family farms are constrained when it comes to buying feed due to economic constraints. In addition, factors such as the study being conducted during a rainy period during the autumn season, the sheep being in the lactation period, and poor indoor comfort and hygiene may have further exacerbated the findings related to poor feeding standards.

The average percentage of minor to severe lameness in sheep at the farm level was 6.49 %. This prevalence was similar to the 5.97 % reported by Marcone et al. (2022), higher than the 4.2 % to 5.2 % reported by Munoz et al. (2019b), but lower than the 7.1 % reported by Phythian et al. (2013) for sheep farms in England and Wales. It is also lower than the prevalence values of 14.5 % and 10.4 % reported by O'Brien et al. (2017) and Kaler and Green (2008), respectively. However, the prevalence of lameness and severe lameness was low (1.3 %) and similar to the 0.40 % reported by Stubsjøen et al. (2022) for Norwegian farms. These results suggest that painful and severe lameness was not a major concern for the farms visited in this study. Nevertheless, the relatively high prevalence of minor lameness indicates a potential lameness risk. Improvements could be achieved through better housing comfort and hygiene, a regular foot health program, good management practices, and enhancing farmers' knowledge and skills regarding hoof care. Lameness is a significant welfare issue and results in economic losses (Green et al., 2012; Vittis and Kaler, 2020; Moschovas et al., 2021). The degree of hoof overgrowth found in this study was 6.27 %, which was higher than that reported by Marcone et al. (2022) (2.20 %) but lower than that reported by Stubsjøen et al. (2022) (11.8 %). In general, there is a moderate prevalence of lameness and overgrown nails on the farms surveyed. This could be caused by poor nutrition, inadequate shelter, turning out to graze, etc. Hoof overgrowth has been suggested as a welfare indicator for assessing the ease of movement in sheep (Richmond et al., 2017). Improper trimming and neglect of treatment can lead to hoof overgrowth, infection, and lameness (Marcone et al, 2022). There are no studies that show a direct relationship between increased hoof overgrowth and decreased mobility. However, a positive correlation has been reported between hoof overgrowth, lameness, and fleece dirtiness (Marcone et al., 2022). Regular foot care is recommended as an improvement strategy for farmers who are dealing with severe foot and hoof problems.

In most farms, the treatment of sick animals by the owner poses a risk factor for animal health management. The average numbers of reforming ewes and dystocia cases were 5.45±1.49 and 2.29±0.44 , respectively. There were no farms that kept regular records of lamb births and lamb deaths. Management practices such as the scanning of pregnant sheep to determine the size of the litter (using ultrasound or related blood biomarkers) were not carried out on any of the farms. According to Goldansaz et al. (2022), early detection of pregnancy in sheep and prediction of the number of lambs that a pregnant ewe will produce have several implications for sheep farmers, particularly in terms of feed management, lambing rate, and sheep and lamb health. On the farms, no panting sheep were observed. This finding may be explained by the fact that the welfare assessments were carried out in the autumn, when ambient temperatures were not high. This argument agrees with the findings of Bodas et al. (2021) and Munoz et al. (2018). However, the mean ratio ( 9.30±2.70 %) of respiratory problems (runny nose and coughing, etc.) in sheep indicates that upper- and lower-respiratory infections occur in sheep from the farms studied. The ranges of the mean proportions of minor and major lesions on body parts such as the eyes, head and/or neck, body, legs, and tail were 0.54–1.50 % and 0.00 %–0.33 %, respectively. In general, these percentages are low. Low stocking densities and reduced social aggression due to grazing may explain this situation. However, the low body lesion scores may also be attributed to the scoring of full-fleeced sheep (Phythian et al., 2019).

The mean proportion of lost ear tags in sheep (or of sheep without earrings) was 5.35±1.72 %. However, the proportion of minor and major ear lesions (ear notches made for numbering purposes, ear tag injuries, skin abrasions and scars) in sheep averaged 9.07 %. This rate was lower than the rate of moderate and severe skin lesions reported by Stubsjøen et al. (2022), which was 14.4 %. The median prevalence of the sum of slight and extensive fleece loss in this study was 20.00 %. In comparison, a median prevalence of bad fleece conditions of 14.25 % was reported for dairy ewes in a study conducted in Italy (Marcone et al., 2022). Poor fleece condition is a useful indicator of sheep welfare as it can be affected by a variety of etiological factors (Richmond et al., 2017). Problems such as itching, fleece loss, and reduced fleece growth in sheep can be caused by external parasites, poor animal care, age, stress, and nutritional imbalances (AWIN, 2015; Taylor, 2012). Fleece quality can be used as an indicator of external parasites affecting sheep welfare as parasitic areas can cause wounds from intense scratching and biting (Llonch et al., 2015). Dual-purpose sheep kept outdoors are more susceptible to micronutrient deficiencies and parasite problems than those kept indoors (Marcone et al., 2022). However, excessive itching was not observed during the physical examination of the animals. Semi-extensively managed sheep, unaccustomed to human presence, were found to be mostly standing and standing very close together during the assessment, which may have prevented excessive itching.

The proportion of sheep that had faecal contamination was large. The prevalence of soiling and extensive soiling with dags extending from the anus to the tail, the upper part of the legs, and down the legs as far as the hocks (scores 4 and 5) was 36.85 % in sheep. This prevalence was higher than those reported by Stubsjøen et al. (2022) (18.8 %) and Phythian et al. (2016) (13.41 %), which were noted to vary with the season. The high level of faecal contamination in this study was attributed to wet and dirty floors in sheep housing facilities and the absence of dry bedding. However, in Europe, fecal soiling in fattening lambs was reported to be either absent or very low (Bodas et al., 2021). This may be an indication that sheep are suffering from parasitic or microbial gastrointestinal problems. According to Zufferey et al. (2021), faecal contamination may be the result of a complex interaction of factors such as gastrointestinal infections or high-quality spring grass. Faecal contamination has been found to be related to the number of eggs in faeces and, hence, to the burden of worms (Broughan and Wall, 2007). The presence of ectoparasites or nutritional problems was also indicated by the proportion of sheep with fleece loss (mean prevalence of 22.94 %). The absence of tail docking and the absence of myiasis were considered to positively affect sheep welfare. No sheep were observed to show any social withdrawal during the welfare assessments. However, the results of the avoidance test (FHAT) showed that the proportion of sheep that did allow handler approach was an average of 11.78 %. This value was lower than the value reported for fattening lambs (the mean for sheep was 0.6) (Bodas et al., 2021). A relatively high proportion of fearful animals may be indicative of the handling conditions or the nature of human–animal interactions. Given that these sheep are semi-extensively reared, they may not be accustomed to frequent handling. As most of these sheep are milked, this may partly reflect the stress associated with milking procedures. Furthermore, it was also suggested that improving human–animal interactions by increasing farmers' knowledge and awareness of animal stress and welfare could prove to be beneficial.

## 5 Conclusion

This study provides important insights into the current status of animal health, behavior, and welfare in family sheep farms operating under the climatic conditions of the Inner West Anatolian region of Türkiye through resource- and animal-based welfare assessments. Our findings highlight the importance of providing high-quality pastures, supplementing with concentrates according to demands, and improving housing quality and animal handling for sheep kept under semi-extensive conditions. The results of this research could make a significant contribution to private-sector or public-policy strategies aimed at improving animal welfare standards in regional sheep production, such as the design of new types of housing and raising awareness regarding the nutritional, health, and behavioral needs of animals.

## Data Availability

The data presented in this study are available from the corresponding author upon reasonable request.
